# Xingpi Yanger Granule combined with conventional medication for the treatment of nocturnal enuresis: a systematic review and meta-analysis of randomized controlled trials

**DOI:** 10.3389/fped.2026.1744259

**Published:** 2026-05-12

**Authors:** Ru-Ping Zhao, Tao Sun, Qiu-Wen Zhang, Mu-Yuan Guo, Shu-Shu Li, Hong Zheng

**Affiliations:** 1 Pediatric Hospital, The First Affiliated Hospital of Henan University of Chinese Medicine, Zhengzhou, China; 2 Faculty of Pediatric, Henan University of Chinese Medicine, Zhengzhou, China; 3 The First Affiliated Hospital of Henan University of Chinese Medicine, Zhengzhou, China; 4 The First Clinical Medical College of Henan University of Chinese Medicine, Zhengzhou, China

**Keywords:** child, meta-analysis, nocturnal enuresis (NE), randomized controlled trials (RCTs), Xingpi Yanger Granule (XPYEG)

## Abstract

**Objective:**

This systematic review aims to determine the clinical efficacy and safety of Xingpi Yanger Granule (XPYEG) in combination with conventional medication for the treatment of Nocturnal Enuresis based on existing data.

**Methods:**

A systematic search was conducted across the China National Knowledge Infrastructure (CNKI), Wanfang Data, China Biomedical Literature Database (CBM), VIP, PubMed, Embase, Cochrane Library, and Web of Science databases to identify relevant RCTs on the auxiliary treatment of NE in children using XPYEG from the inception of each database up to August 2025. The risk of bias in the included RCTs was assessed using the Cochrane Risk of Bias tool (ROB2). Meta-analysis was performed using Review Manager, and the sensitivity analysis was conducted using Stata 18 software. The quality of evidence was evaluated with the GRADE approach. Furthermore, Trial Sequential Analysis (TSA) was employed to determine the required sample size and to verify the reliability of the findings. The review protocol was registered and published on PROSPERO (CRD420251156842).

**Results:**

This study included 12 RCTs involving 1,138 patients. Compared with conventional therapy alone, the combination of XPYEG significantly improves core symptoms, which is clinically significant. Core Symptom and Functional Outcomes: Compared with the control group, adjunctive XPYEG therapy resulted in a notable reduction in the frequency of NE [MD = 1.55, 95% CI (0.66, 2.43), *P* < 0.00001], a metric of direct relevance to quality of life. This symptomatic relief was supported by overall response rate [RR = 1.25, 95% CI (1.18, 1.32), *P* < 0.00001] and objective functional improvements, including a significant increase in age-expected bladder capacity [MD = 41.31, 95% CI (36.31, 46.32), *P* < 0.00001]. Long-term Trajectory and Safety: The combined regimen was further associated with a substantially lower risk of symptom recurrence [RR = 0.30, 95% CI (0.18, 0.49), *P* < 0.00001] and a reduced incidence of adverse reactions [RR = 0.35, 95% CI (0.15, 0.80), *P* < 0.00001], underscoring its potential as a sustainable and well-tolerated adjunctive strategy. Biochemical Correlates: Accompanying these clinical benefits, favorable modulations were observed in antidiuretic hormone (AVP) levels [MD = 6.40, 95% CI (5.18, 7.62), *P* < 0.00001], urinary osmolality [MD = 44.61, 95% CI (38.72, 50.48), *P* < 0.00001] and related second messenger pathways (cAMP, cGMP, and BVWI). While statistically significant, these findings serve primarily as supportive mechanistic rationale rather than direct indicators of patient-centered benefit.

**Conclusion:**

Xingpi Yanger Granule may improve overall response, moderately reduce urinary frequency, and increase bladder capacity, while also lowering the recurrence rate and the incidence of adverse events. However, given that the evidence is limited in scope and rated as low to moderate quality, current conclusions should be interpreted with caution. Well-designed, large-scale, multicenter randomized controlled trials are needed to validate these potential benefits.

**Systematic Review Registration:**

https://www.crd.york.ac.uk/PROSPERO/, PROSPERO CRD420251156842.

## 1 Introduction

Nocturnal enuresis (NE) refers to the involuntary urination that occurs during nighttime sleep in children aged five and older or adolescents, at least once a month, for a period of three months or longer, and constitutes a common condition (1–4). Epidemiological studies indicate that the prevalence of NE among preschool children in Asia ranges from approximately 21.0%–27.8%, while the prevalence among school-aged children is 6.9% to 11.2%. In Europe and America, the prevalence ranges from 3.8% to 18.9% (2, 3). NE not only causes sleep disturbances in children and affects their physical development, but also often leads to psychological issues such as low self-esteem and anxiety (4, 5). Approximately 15% of affected children may experience spontaneous remission as they grow older, but 1%–2% of cases persist into adulthood (6), placing a continuous physical and emotional burden on the children and their families. Currently, first-line treatment options for NE include psychological and behavioral interventions, conventional pharmacological treatments such as desmopressin, and enuresis alarms (7, 8). However, these therapies are primarily aimed at symptom control and have significant limitations: medications may cause adverse reactions, some children may develop treatment resistance, and there is a high rate of relapse after discontinuation (9–11). Therefore, there is an urgent clinical need for safe, sustainable adjunctive treatment strategies to address the shortcomings of existing regimens.

Traditional Chinese medicine has a long history of use in the treatment of NE. Its multi-targeted, holistic regulatory approach not only helps alleviate clinical symptoms but also offers the advantages of long-lasting efficacy, low recurrence rates, and good safety (12). Xingpi Yanger Granule (XPYEG) is a traditional Chinese patent medicine approved by the National Medical Products Administration (Approval No. Z20025415) (13), with main ingredients including Yidian Hong *(Emilia sonchifolia*), Maoda Dingcao (*Gerbera piloselloides*), Shanzhi Cha (*Pittosporum glabratum Lindl*) and Zhizhu Xiang (*Valeriana jatamansi Jones*) (14, 15). Modern pharmacological studies indicate that it possesses various biological activities, including anti-inflammatory, sedative, antispasmodic, and neural modulatory effects (16–20), which are potentially related to the multifactorial pathophysiological mechanisms of NE. In clinical practice, XPYEG is often used as an adjunctive medication in combination with conventional medical treatment to treat conditions such as enuresis, night sweats, and loss of appetite caused by spleen deficiency in children. Its granule formulation is convenient for oral administration, well-tolerated by children, and associated with few and mild adverse reactions-primarily mild gastrointestinal discomfort-demonstrating good overall safety (21, 22).

Although several randomized controlled trials have preliminarily explored the efficacy of XPYEG as an adjuvant therapy for NE (13, 14, 21), there is currently a lack of high-quality systematic evidence summaries. Most existing studies are limited by their single-center design and small sample sizes, and they lack sufficient evaluation of long-term safety, recurrence rates, and mechanisms of action. Therefore, this study aims to comprehensively evaluate the clinical efficacy and safety of XPYEG combined with conventional medication for NE through a systematic review and meta-analysis, and to preliminarily explore its potential mechanisms of action, with the goal of providing more reliable evidence-based guidance for clinical practice.

## 2 Methods

### 2.1 Study registration

This study was conducted in strict adherence to the Preferred Reporting Items for Systematic Reviews and Meta-Analyses (PRISMA 2020) guidelines (23). It has been registered on the PROSPERO international prospective systematic review registry (Registration number: CRD420251156842).

All XPYEG used in the clinical trials included in this study were manufactured under approval from the China National Medical Products Administration, with batch number: Z20025415.

### 2.2 Literature search

Two researchers (TS and RZ) independently performed the systematic literature search in eight Chinese and English electronic databases: PubMed, Web of Science, Embase, the Cochrane Library, CNKI, Wanfang, VIP, and SinoMed. The search covered the period from each database's inception to August 2025. Supplementary manual searches were conducted using the China Clinical Trials Registry and by scanning the reference lists of included studies and relevant reviews. The search strategy combined Medical Subject Headings (MeSH) terms with free-text keywords (e.g., “enuresis”, “nocturnal enuresis”, “bedwetting”, “Xingpi Yanger”, “Xingpi Yanger Granule”). Full search details are provided in Supplementary Material. Inclusion and Exclusion Criteria

#### 2.3.1 Inclusion criteria

Based on the PICOS framework, the inclusion criteria for this study are as follows:

1. Population (P): Children or adolescents aged 5 years and above, but not exceeding 16 years of age, diagnosed with enuresis, irrespective of gender or ethnicity.
2. Intervention and Control Groups (I/C): Patients in the control group received first-line treatment for nocturnal enuresis as recommended by contemporary guidelines, primarily including psychological and behavioral interventions, conventional pharmacotherapy (e.g., desmopressin), and bedwetting alarms or a combination of both. This was supplemented by routine symptomatic management (such as dose adjustment, temporary discontinuation, or localized care) to address associated adverse reactions including headaches, hyponatremia, or skin irritation. The intervention group received XPYEG in addition to the same control regimen, administered orally three times daily at 4 grams per dose (two sachets) dissolved in water. Specific treatment protocols for each trial are detailed in Table 1.
3. Outcome Measures (O): The primary efficacy endpoint is clinical treatment efficacy rate. In the included trials, the ORR was defined as the proportion of patients who achieved a complete response (a ≥90% reduction in weekly bedwetting episodes) or a partial response (a 50%–90% reduction). All trials adhered to the standards of the Chinese Clinical Guidelines for Pediatric Enuresis, thereby allowing for a valid pooled analysis. Secondary outcome measures include: (1) Clinical symptom or functional assessment indicators: enuresis frequency, bladder capacity (All included studies reporting this outcome were assessed via transabdominal ultrasound at the time the child first felt the urge to urinate), arousal threshold, bladder wall weight index (BVWI), urine osmolality; (2) Biochemical indicators: cyclic adenosine monophosphate (cAMP), arginine vasopressin (AVP/ADH) (Blood samples were collected between 6:00 and 8:00 a.m. in a fasting state), cyclic guanosine monophosphate (cGMP), cAMP/cGMP ratio [The determination of the aforementioned biochemical indicator levels was primarily conducted using enzyme-linked immunosorbent assay (ELISA) kits, adhering to standard operating procedures.]; (3) Adverse drug reactions (ADRs) incidence, including gastrointestinal reactions, hepatic/renal injury, and recurrence rate.
4. Study Type (S): Research design restricted to RCTs, without limitations on blinding status, publication type, or language.

**Table 1:** The characteristics of all included studies.

Author and reference	Sample size		Mean age (years)		Interventions		Treatment duration (week)		Outcomes
	T	C	T	C	T	C	T	C	
Fu et al. (13)	33	33	10.34 ± 2.52	10.32 ± 2.55	C + XPYEG	DDAVP	8	8	①②③④⑤⑥⑦⑧⑭
Hao et al. (14)	46	46	9.62 ± 2.17	9.58 ± 2.11	C + XPYEG	Racanisodamine + Vit B1 + Oryzanol + Meclofenoxate	4	4	①③④⑤⑥⑦⑨⑪⑫
Lang et al. (21)	87	70	-	-	C + XPYEG	Psychotherapy	8	8	①⑫
Li et al. (24)	44	44	6.98 ± 3.42	7.63 ± 2.31	C + XPYEG	Racanisodamine + Vit B1 + Oryzanol + Meclofenoxate	6	6	①⑪
Lin et al. (25)	50	50	8.2 ± 0.4	8.2 ± 0.4	C + XPYEG	DDAVP	12	12	①⑫
Wan et al. (26)	74	74	6.81 ± 1.01	6.77 ± 1.01	C + XPYEG	Meclofenoxate	4	4	①②③⑥⑨⑩⑫⑬
Wang et al. (27)	41	41	7.49 ± 1.71	7.55 ± 1.78	C + XPYEG	Racanisodamine + Vit B1 + Oryzanol + Meclofenoxate	4	4	①③⑨⑪⑫
Wang et al. (16)	45	45	7.43 ± 1.62	7.27 ± 1.45	C + XPYEG	DDAVP	12	12	①②③
Yang et al. (17)	58	58	8.78 ± 1.63	8.01 ± 1.65	C + XPYEG	DDAVP	12	12	①
Yi et al. (18)	48	48	6.98 ± 1.04	6.75 ± 1.13	C + XPYEG	DDAVP	6	6	①⑥⑩
Yu et al. (19)	51	46	7.62 ± 2.53	7.74 ± 2.09	C + XPYEG	Racanisodamine + Vit B1 + Oryzanol + Meclofenoxate	4	4	①②③④⑤⑥⑧⑨⑪⑫
Zhou et al. (20)	45	45	7.1 ± 1.5	6.8 ± 1.2	C + XPYEG	DDAVP	12	12	①⑥⑩

T, treatment group; C, control group.

Interventions: XPYEG, Xingpi Yanger Granules; DDAVP, Desmopressin Acetate Tablets; Meclofenoxate, Meclofenoxate Hydrochloride Capsules; Racanisodamine + Vit B1 + Oryzanol + Meclofenoxate, Racanisodamine Tablets + Vitamin B1 Tablets + Oryzanol Tablets + Meclofenoxate Hydrochloride Capsules; Psychotherapy.

Outcomes: ① Overall Response Rate; ② Reduction in Arousal Threshold; ③ Increase in Bladder Capacity; ④ Reduction in BVWI; ⑤Increase in cAMP; ⑥ Increase in AVP Levels; ⑦ cGMP reduction; ⑧ Increase in cAMP/cGMP Ratio; ⑨ Reduction in Enuresis Frequency; ⑩ Increase in Urine Osmolarity; ⑪ Incidence of adverse drug reactions (ADRs); ⑫ Recurrence Rate; ⑬ sleep-wake disorder scores; ⑭ 24-h urinary 17-OH levels.

#### 2.3.2 Exclusion criteria

Based on the PICOS framework, the exclusion criteria for this study are as follows:

1. Participants (P): Individuals with secondary enuresis or those presenting with other severe complications;
2. Interventions and controls (I/C): Research on the concurrent use of other Chinese herbal compound preparations or traditional Chinese medical therapies during intervention;
3. Outcomes (O): Studies with incomplete data reporting or where complete data could not be extracted; duplicated publications;
4. Study design (S): Non-RCTs, including animal studies, *in vitro* research, reviews, case reports, and letters to the editor.

### 2.4 Data extraction

Two researchers (R.Z. and T.S.) independently screened the literature according to predefined search strategies and inclusion/exclusion criteria. Initial screening was conducted based on titles and abstracts, followed by full-text review for final selection and data extraction. All extracted information was recorded using an Excel spreadsheet, including key details such as first author, publication year, diagnostic criteria, sample size, randomized methods, intervention and control measures, outcome measures, and results data. Following extraction, the two researchers cross-checked their findings. Disagreements were resolved through joint discussion and negotiation. Where consensus remained unattainable, a third researcher (Q.Z.) was consulted for arbitration to ensure the objectivity and impartiality of the data extraction process.

### 2.5 Risk of bias assessment

Two assessors (M.G. and S.L.) independently evaluated the risk of bias in included studies using the ROB 2.0 risk of bias assessment tool, with cross-checking performed. Disagreements were resolved by a third assessor (R.Z.), who made the final determination. The assessment covered the following five domains: (1) randomized process; (2) Deviations from the intervention protocol; (3) Missing outcome data; (4) Outcome measurement methods; (5) Selective reporting of results. According to the ROB 2 criteria, risk of bias assessments were classified as “low”, “some concerns”, or “high”. This grading was based on responses to a series of signal questions within each domain, with possible answers including: “Yes” (Y), “Probably yes” (PY), “Probably no” (PN), “No” (N), and “No information” (NI).

### 2.6 Statistical analysis

All statistical analyses were conducted using Cochrane Review Manager (RevMan) 5.4 software. For dichotomous variables, effect sizes were expressed as risk ratios (RR) with 95% confidence intervals (CI). Continuous variables were represented by mean differences (MD) with 95% CI. Heterogeneity between studies was assessed using *χ*² tests (set at a significance level of *P* < 0.10) and the *I*^2^ statistic. When significant heterogeneity is present (*P* < 0.10 and *I*^2^ ≥ 50%), a random-effects model is employed for meta-analysis; otherwise, a fixed-effects model is used. Differences were considered statistically significant at *P* < 0.05 (28).

### 2.7 Subgroup analysis and sensitivity analysis

To explore heterogeneity among included studies, subgroup analyses were conducted according to a pre-specified protocol. These analyses aim to evaluate the impact of different treatment cycles (4–6 weeks and 8–12 weeks) within the treatment group on the outcome measures. Additionally, to test the robustness of pooled results and identify potential sources of heterogeneity, sensitivity analyses were conducted using STATA 18.0 software. This involved sequentially excluding individual studies for validation.

### 2.8 Quality of evidence

Furthermore, the GRADEpro GDT online tool (accessible at: http://www.gradepro.org/) (29) was employed to assess the quality of evidence for outcome measures. All RCTs were initially assigned a high certainty grade. Evidence certainty was subsequently assessed across five domains: risk of bias, inconsistency, indirectness, imprecision, and publication bias. Evidence was graded as high certainty if no downgrading factors were present; moderate certainty if one downgrading factor existed; low certainty if two downgrading factors were present; and three or more downgrading factors resulted in a very low level. Detailed results are presented in the appendix.

### 2.9 Trial sequence analysis (TSA)

To minimize the risk of erroneous conclusions arising from random errors in the meta-analysis, a trial sequence analysis (TSA) (39) was further conducted. TSA employs an alpha-spending function to set statistical significance thresholds, thereby controlling Type I error (false positive) risk, while utilizing a beta-spending function and constructing an ineffectiveness boundary to control Type II error (false negative) risk. The *Z*-statistic is calculated by dividing the logarithm of the pooled effect size of the interventions by its standard error. When the cumulative Z curve breaches the Trial Sequential Monitoring boundary or the power boundary, it indicates that the current evidence's sample size is sufficient to detect the anticipated intervention effect, and future trials are unlikely to alter the existing conclusion (30). Accordingly, we employed Trial Sequential Analysis software v.0.9.5.10 Beta (Copenhagen Centre for Clinical Trials, Denmark) to assess whether the overall sample size met statistical significance requirements. The analysis settings were as follows: boundary type was two-sided, Type I error probability was set at 5%, and test power was set at 80%. The relative risk reduction rate and control group event rate were both set based on the results of the current meta-analysis. By calculating the cumulative *Z*-statistic and the required information size, this study aimed to make a clear inference about the target effect size.

## 3 Results

### 3.1 Literature search results and study characteristics

**Figure 1:**
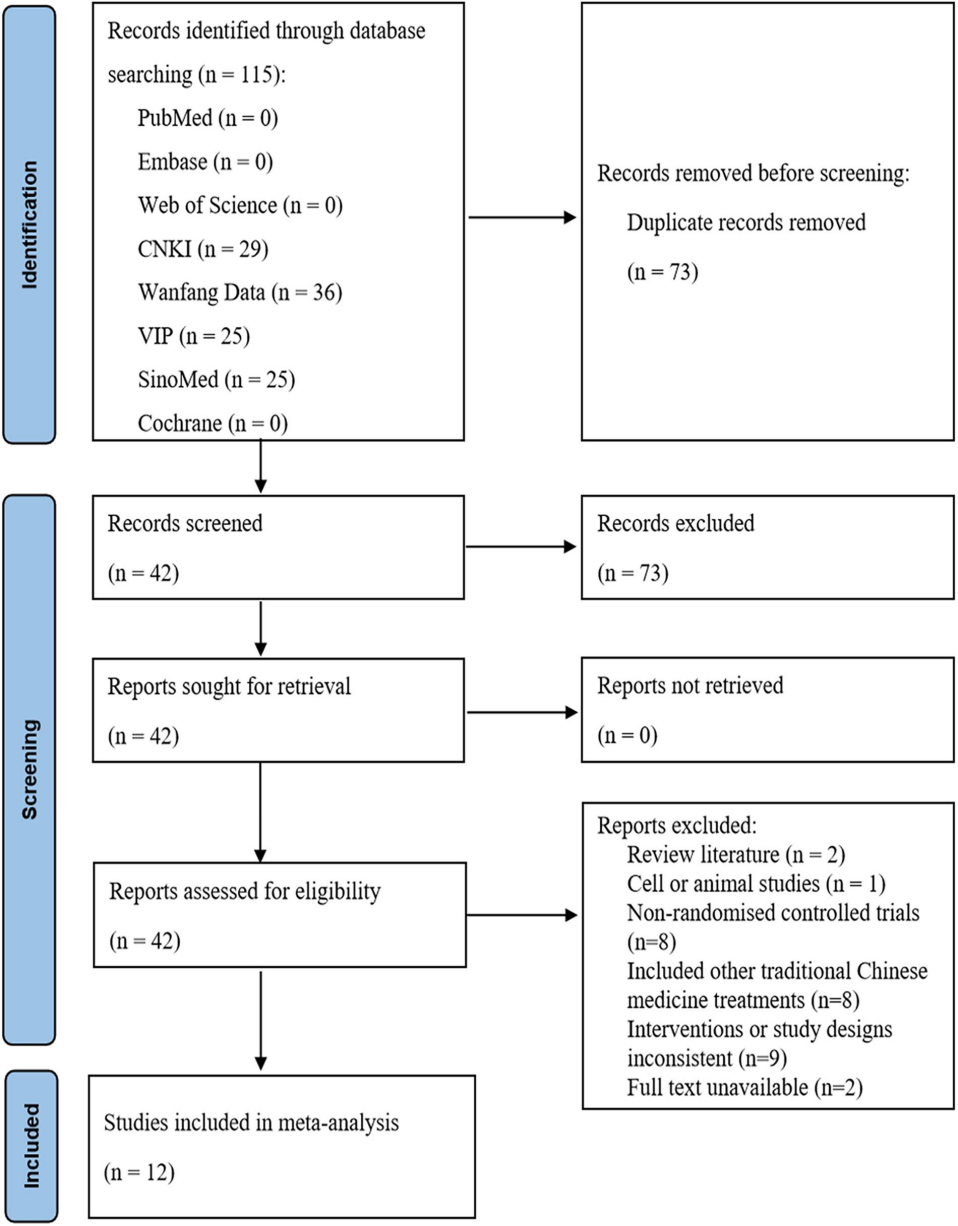
Flow diagram of the literature search.

The initial search yielded 115 relevant publications. After removing 73 duplicates using NoteExpress, 42 studies remained. Preliminary screening of titles and abstracts excluded 19 studies that did not meet the inclusion criteria. Full-text review and assessment of the remaining 23 studies resulted in the exclusion of 11, leaving 12 studies that met inclusion criteria and were ultimately included in the analysis (Figure 1). The studies spanned publication dates from 2012 to 2024, were all conducted in China, and involved a total of 1,138 patients. Sample sizes ranged from 66 to 157 participants. Key study characteristics and specific intervention details were summarized in Table 1.

### 3.2 Risk of bias assessment

**Figure 2:**
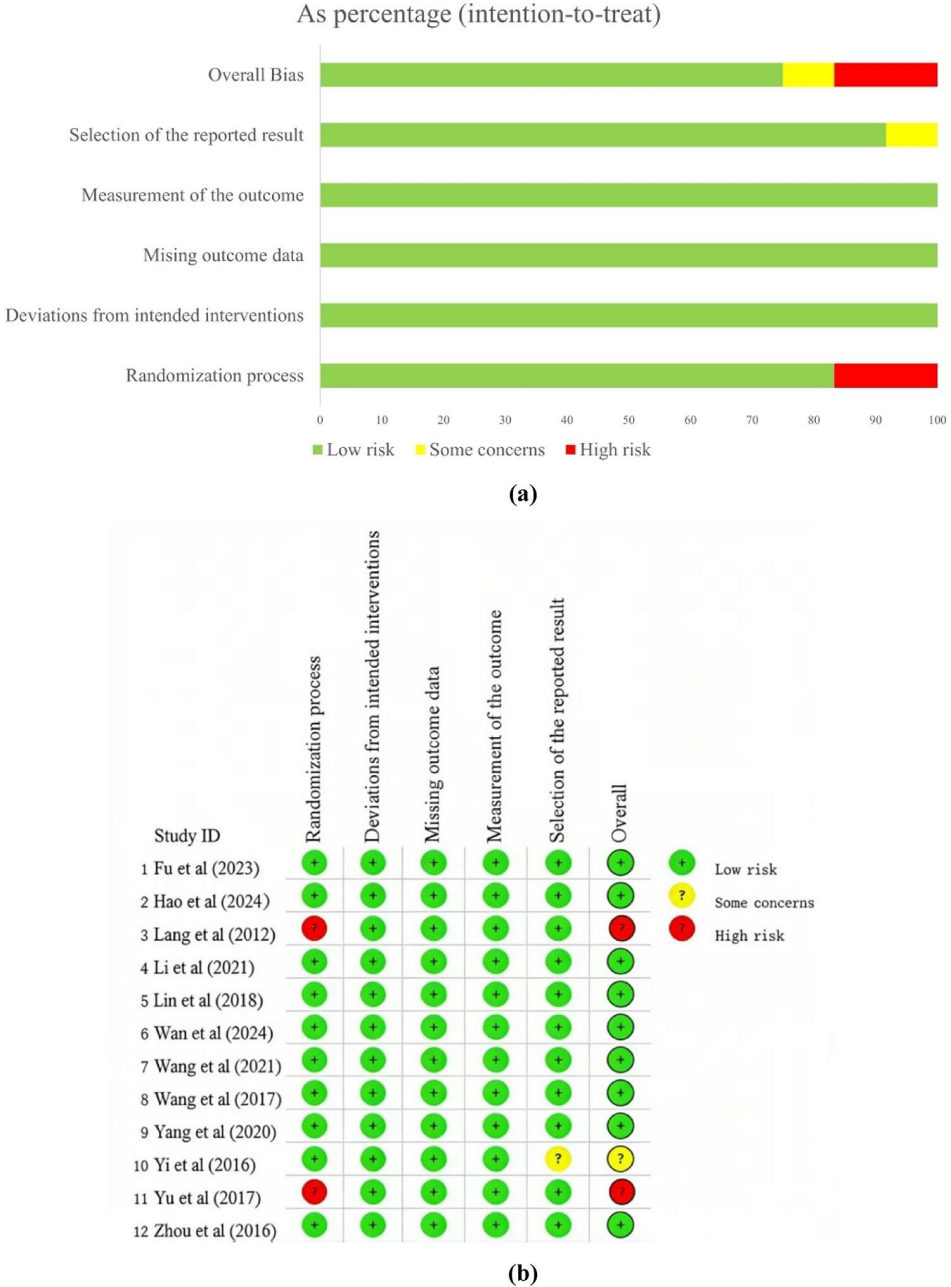
Risk of bias of included study. **(a)** risk of bias summary; **(b)** risk of bias graph.

In summary, across all included trials, nine studies were rated as having a “low risk of bias”, two studies were rated as having a “high risk of bias” due to potential incomplete randomization, and one study was rated as having an “uncertain risk of bias” due to selective reporting of results. Detailed risk of bias assessment results are presented in Figure 2.

### 3.3 Primary outcome measures

#### 3.3.1 Overall response rate

**Figure 3:**
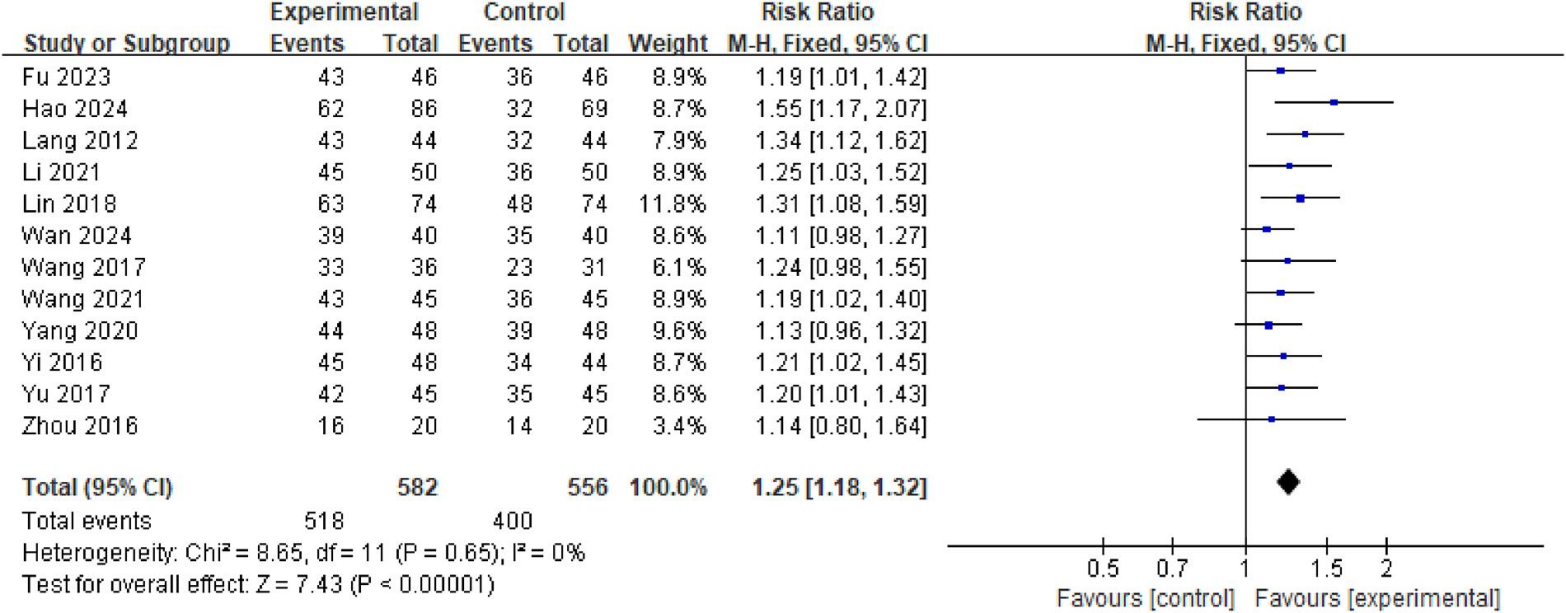
Forest plot of ORR.

**Figure 4:**
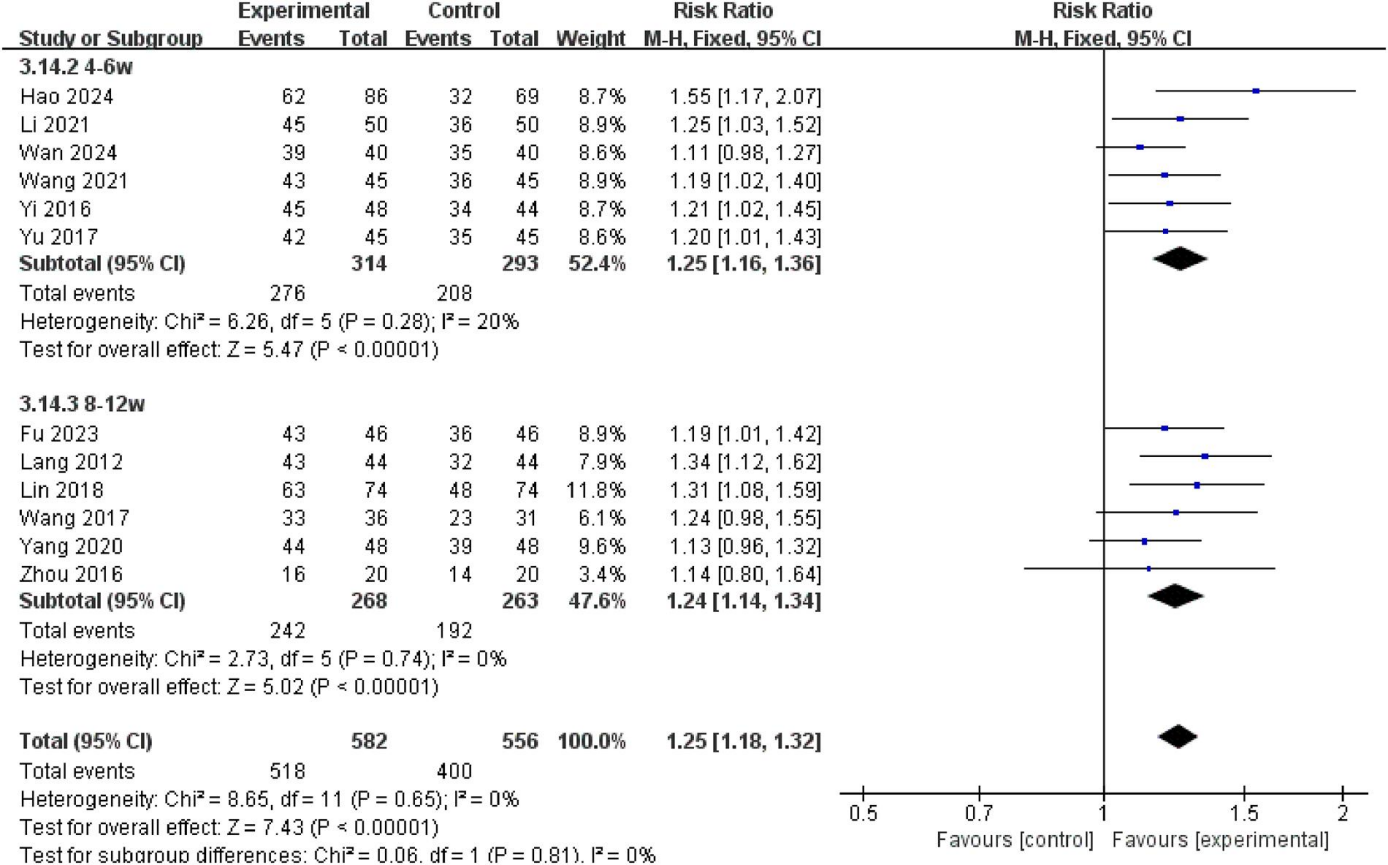
Forest plot of subgroup analysis of ORR.

Twelve studies reported ORR, involving a total of 1,138 patients. Heterogeneity analysis revealed no significant heterogeneity between studies (*P* = 0.65, *I*^2^ = 0%), thus permitting meta-analysis using a fixed-effect model. Meta-analysis results demonstrated a statistically significant higher overall response rate in the treatment group compared to the control group [RR = 1.25, 95% CI: (1.18, 1.32), *P* < 0.00001] (Figure 3). To evaluate the efficacy of XPYEG adjuvant therapy for NE across different treatment durations, we conducted subgroup analyses comparing the 4–6 week and 8–12 week treatment groups. The 4–6 week group (RR = 1.25, 95% CI: 1.16–1.36, *p* < 0.00001) and the 8–12 week group (RR = 1.24, 95% CI: 1.14–1.34, *p* < 0.00001) showed no significant heterogeneity between groups (*I*^2^ = 0%) (Figure 4).

### 3.4 Secondary outcomes

#### 3.4.1 Reduction in enuresis frequency

**Figure 5:**
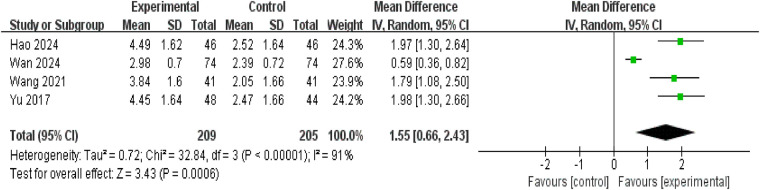
Forest plot of reduction in enuresis frequency.

Four studies reported reductions in enuresis frequency before and after treatment, involving a total of 414 participants (intervention group: 209; control group: 205). Significant heterogeneity existed between studies (*P* < 0.00001, *I*^2^ = 91%), necessitating analysis using a random-effects model. Results indicated a greater reduction in enuresis frequency in the intervention group compared to the control group [MD = 1.55, 95% CI: (0.66, 2.43), *P* = 0.0006] (Figure 5). Sensitivity analysis using the “exclusion of individual studies” method identified Wan Juan 2024 (26) as the primary source of heterogeneity. Excluding this study completely eliminated heterogeneity (*P* = 0.91, *I*^2^ = 0%) while maintaining stable observational results [MD = 1.92, 95% CI: (1.52, 2.31), *P* < 0.00001].

#### 3.4.2 Increase in bladder capacity

**Figure 6:**
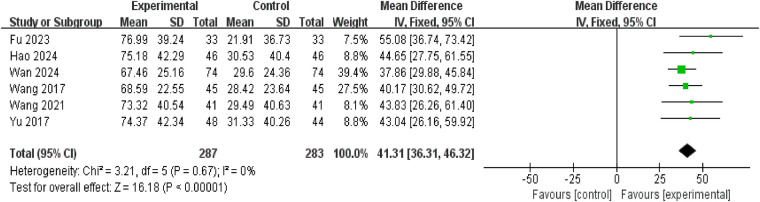
Forest plot of increase in bladder capacity.

Six studies involving 570 patients (287 in the intervention group and 283 in the control group) reported changes in bladder capacity. As no significant heterogeneity was detected (*P* = 0.67, *I*^2^ = 0%), a fixed-effect model was employed for analysis. Results indicated that the increase in bladder capacity was greater in the treatment group than in the control group (mean difference = 41.31, 95% confidence interval: 36.31–46.32; *P* < 0.00001) (Figure 6).

#### 3.4.3 Reduction in arousal threshold

**Figure 7:**
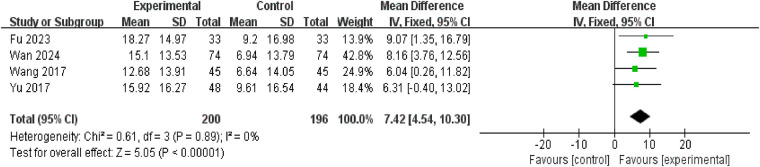
Forest plot of reduction in arousal threshold.

Four studies reported arousal thresholds, involving a total of 396 participants (200 in the intervention group and 196 in the control group). No significant heterogeneity was observed (*P* = 0.89, *I*^2^ = 0%), permitting meta-analysis using a fixed-effects model. Results demonstrated a statistically significant greater reduction in arousal thresholds in the treatment group compared to the control group [mean difference = 7.42, 95% confidence interval: (4.54, 10.30), *P* < 0.00001] (Figure 7).

#### 3.4.4 Reduction in BVWI

**Figure 8:**
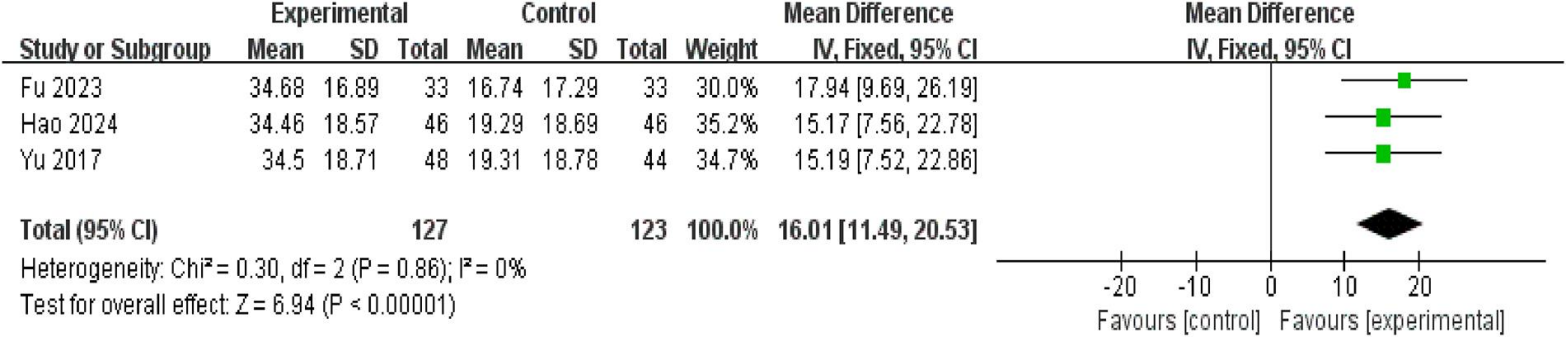
Forest plot of reduction in BVWI.

Three studies reported reductions in pediatric BVWI, involving 250 patients (127 in the intervention group, 123 in the control group). Heterogeneity testing revealed no significant variation between studies (*P* = 0.86, *I*^2^ = 0%), thus permitting meta-analysis using a fixed-effect model. Results indicated that the reduction in BVWI was greater in the treatment group than in the control group [mean difference = 16.01, 95% confidence interval: (11.49, 20.53), *P* < 0.00001], demonstrating statistical significance (Figure 8).

#### 3.4.5 Increase in cAMP

**Figure 9:**
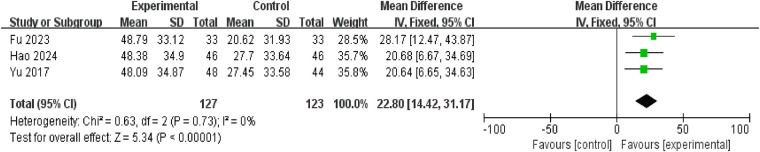
Forest plot of increase in cAMP.

Three studies reported changes in pediatric cAMP levels, involving 250 patients (127 in the intervention group, 123 in the control group). No heterogeneity was observed between studies (*P* = 0.73, *I*^2^ = 0%), thus a fixed-effect model was employed for meta-analysis. Results demonstrated that cAMP levels in the treatment group were higher than those in the control group [mean difference = 22.80, 95% confidence interval: (14.42, 31.17), *P* < 0.00001] (Figure 9).

#### 3.4.6 Increase in AVP level

**Figure 10:**
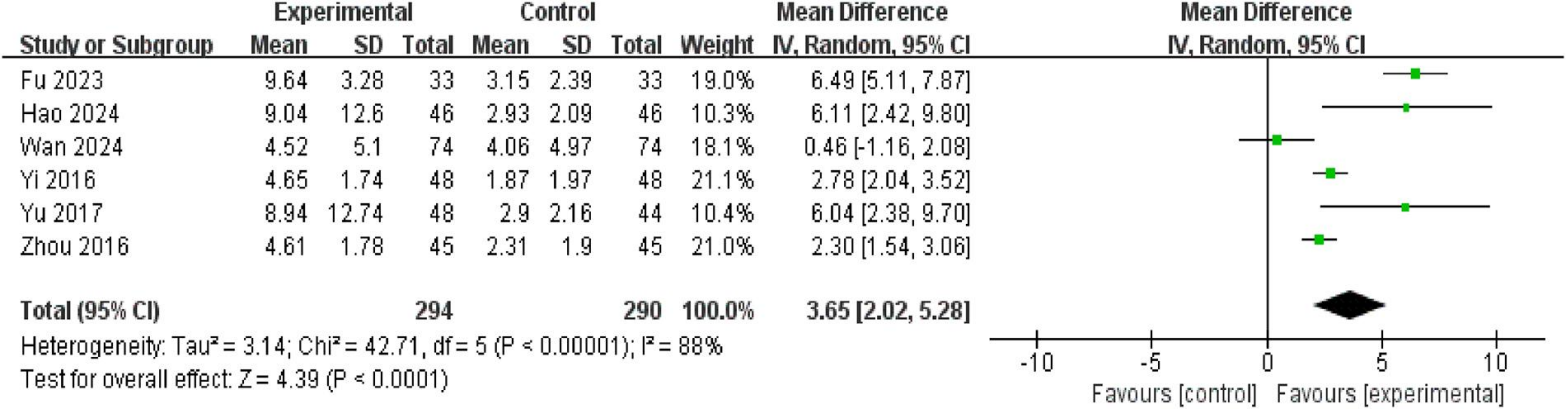
Forest plot of increase in AVP.

Six studies, involving a total of 584 participants (294 in the intervention group and 290 in the control group), reported changes in patients' AVP levels. Four of these studies explicitly stated that blood samples were collected between 6:00 and 8:00 a.m. in a fasting state, while the other two did not report the sampling time. The studies exhibited considerable heterogeneity (*P* < 0.00001, *I*^2^ = 88%), necessitating analysis using a random-effects model. Results indicated that the increase in AVP levels was greater in the treatment group than in the control group (mean difference = 3.60, 95% confidence interval: 2.02–5.28; *P* < 0.00001) (Figure 10).

#### 3.4.7 cGMP reduction

**Figure 11:**
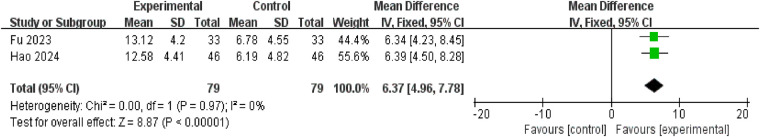
Forest plot of cGMP reduction.

Two studies reported changes in pediatric cGMP levels before and after treatment, involving 158 patients (79 in the intervention group, 79 in the control group). Heterogeneity testing revealed no significant variation between studies (*P* = 0.97, *I*^2^ = 0%), thus a fixed-effect model was employed for meta-analysis. Results indicated that the increase in cGMP was higher in the treatment group than in the control group [MD = 6.37, 95% CI: (4.96, 7.78), *P* < 0.00001], demonstrating a statistically significant difference (Figure 11).

#### 3.4.8 Increase in cAMP/cGMP ratio

**Figure 12:**
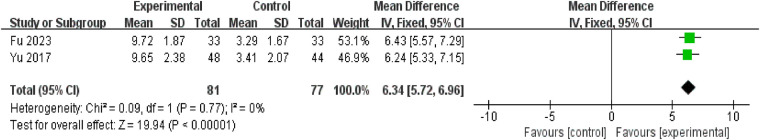
Forest plot of increase in cAMP/cGMP ratio.

Two studies reported elevated levels of cAMP/cGMP before and after treatment, involving a total of 158 subjects (81 in the observation group and 77 in the control group). No significant heterogeneity was observed (*P* = 0.77, *I*^2^ = 0%), permitting analysis using a fixed-effect model. Results demonstrated a higher increase in cAMP/cGMP levels in the observation group compared to the control group [MD = 6.34, 95% CI: (5.72, 6.96), *P* < 0.00001] (Figure 12).

#### 3.4.10. Increase in urine osmolarity

**Figure 13:**
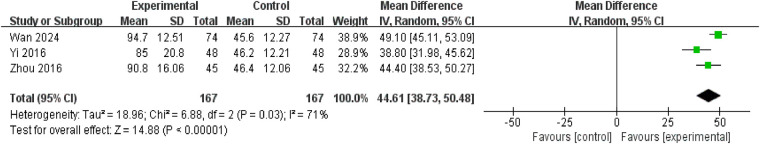
Forest plot of increase in urine osmolarity.

Three studies reported increases in urinary osmolality before and after treatment in 334 subjects (167 per group). Results indicated a greater increase in the treatment group compared to the control group [MD = 44.61, 95% CI: (38.73, 50.48), *P* < 0.00001] (Figure 13). Heterogeneity analysis revealed significant heterogeneity (*P* = 0.03, *I*^2^ = 71%), necessitating random-effects model analysis. Sensitivity analysis using the “single-study exclusion method” identified Wan Juan 2024 (26) as the primary source of heterogeneity. Exclusion of this study reduced heterogeneity to the lowest level (*P* = 0.22, *I*^2^ = 33%) while maintaining stable observational results [MD = 41.88, 95% CI: (36.42, 47.34), *P* < 0.00001].

#### 3.4.11. Incidence of adverse drug reactions (ADRs)

**Figure 14:**
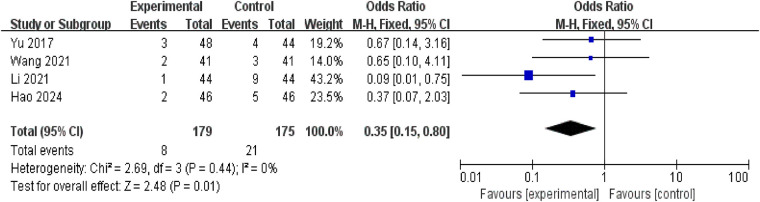
Forest plot of ADRs.

Four studies reported adverse reactions during treatment, involving 354 patients (179 in the treatment group and 175 in the control group). The adverse reactions reported in the study primarily included gastrointestinal discomfort (nausea, vomiting, abdominal pain), headache, and impaired liver and kidney function. Serious adverse events were relatively uncommon. Specifically, the control group reported nine cases of nausea, five cases of vomiting, six cases of gastric discomfort, six cases of mild abdominal pain, and one case of headache. The treatment group reported three cases of nausea, one case of vomiting, one case of gastric discomfort, and three cases of mild abdominal pain. No significant heterogeneity was observed between studies (*P* = 0.44, *I*^2^ = 0%), permitting meta-analysis using a fixed-effect model. Results indicated a lower incidence of adverse reactions in the treatment group compared to the control group [RR = 0.35, 95% CI: (0.15, 0.80), *P* = 0.01], representing a statistically significant difference (Figure 14).

#### 3.4.12. Recurrence rate

**Figure 15:**
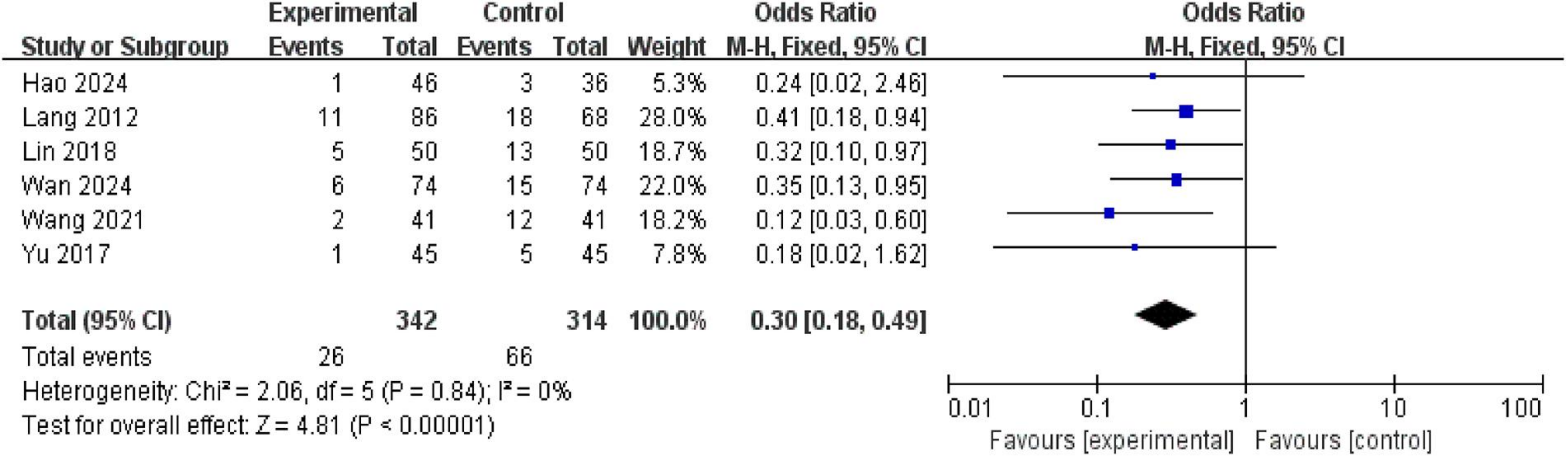
Forest plot of recurrence rate.

Six studies reported recurrence rates for NE, involving a total of 656 patients (342 in the intervention group and 314 in the control group). No significant heterogeneity was observed between studies (*P* = 0.84, *I*^2^ = 0%), permitting meta-analysis using a fixed-effect model. Results demonstrated a statistically significant reduction in recurrence rate within the treatment group compared to the control group [RR = 0.30, 95% CI (0.18, 0.49), *P* < 0.00001] (Figure 15).

#### 3.4.13. Other outcome measures

Certain outcome measures were subject only to descriptive analysis, as they appeared solely in individual study reports or lacked complete raw data. One study (24) reported sleep-wake disturbance scores, with the treatment group exhibiting lower scores than the control group, a difference being statistically significant (*P* < 0.05). Another study (13) documented changes in 24-h urinary 17-hydroxyprogesterone levels before and after treatment: no significant difference existed between groups at baseline (*P* = 0.097). Post-treatment, the treatment group exhibited a greater increase in levels than the control group (*P* < 0.001).

### 3.5 Sensitivity analysis

**Figure 16:**
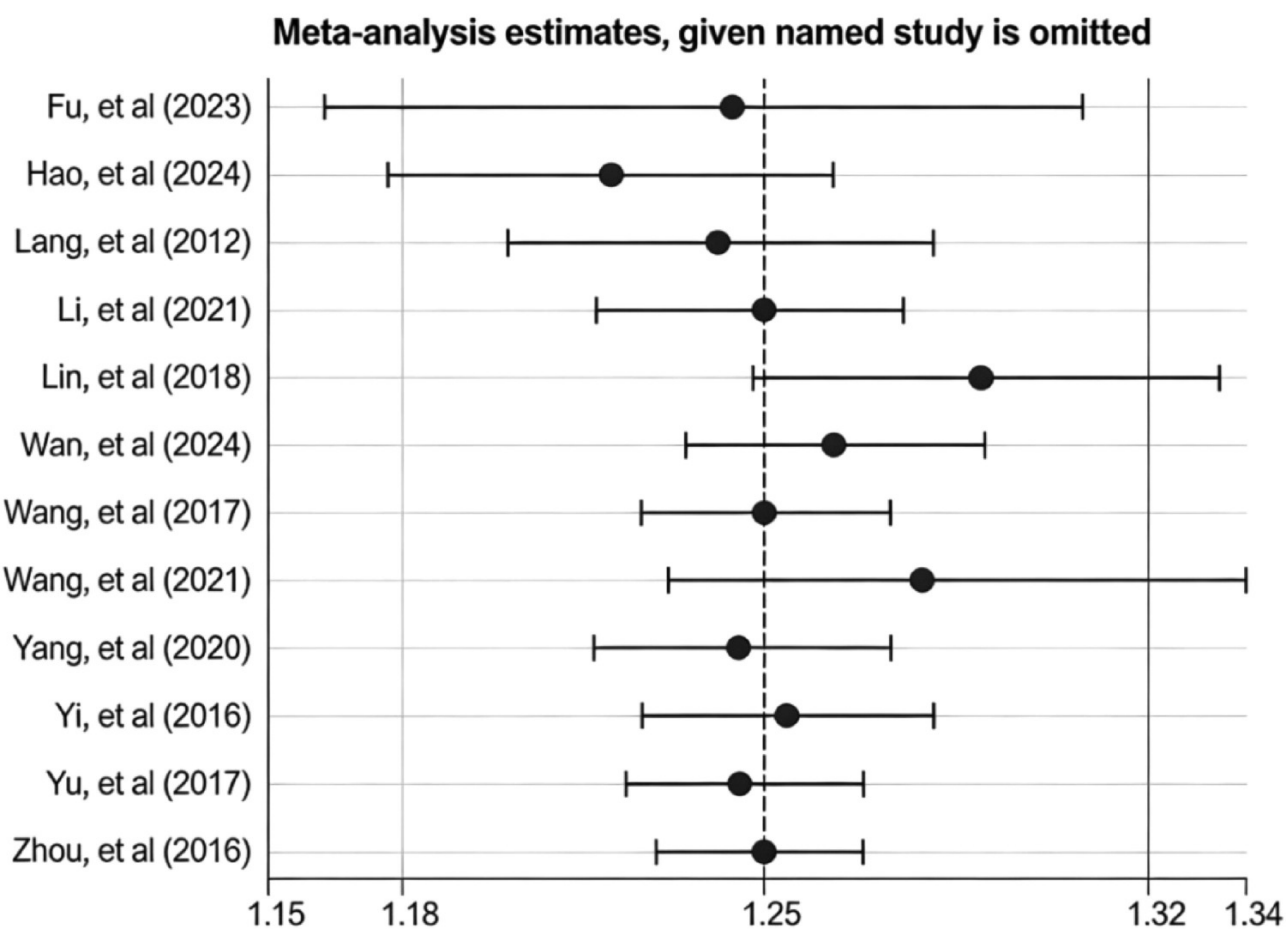
Sensitivity analyses of primary outcomes ORR.

To evaluate the robustness of the primary outcomes (ORR, Bladder Capacity, Recurrence Rate), we performed a sensitivity analysis by sequentially excluding individual studies. The pooled effect sizes remained stable throughout this process, confirming the robustness of our meta-analysis findings. The results for the primary outcomes are shown in Figure 16, and those for other outcomes are provided in Supplementary Figure S1.

### 3.6 Evidence quality grading results

Using the GRADEpro framework, the overall confidence in the evidence was assessed as moderate for the outcomes of ORR, BVWI, and recurrence rate. For all other outcomes, the evidence was considered low or very low quality. Reasons for downgrading included the risk of bias associated with lack of blinding, high heterogeneity between studies, imprecision (wide confidence intervals), and the limited number of available studies. The complete grading for each outcome is detailed in Supplementary Table S1.

### 3.7 Trial sequence analysis

**Figure 17:**
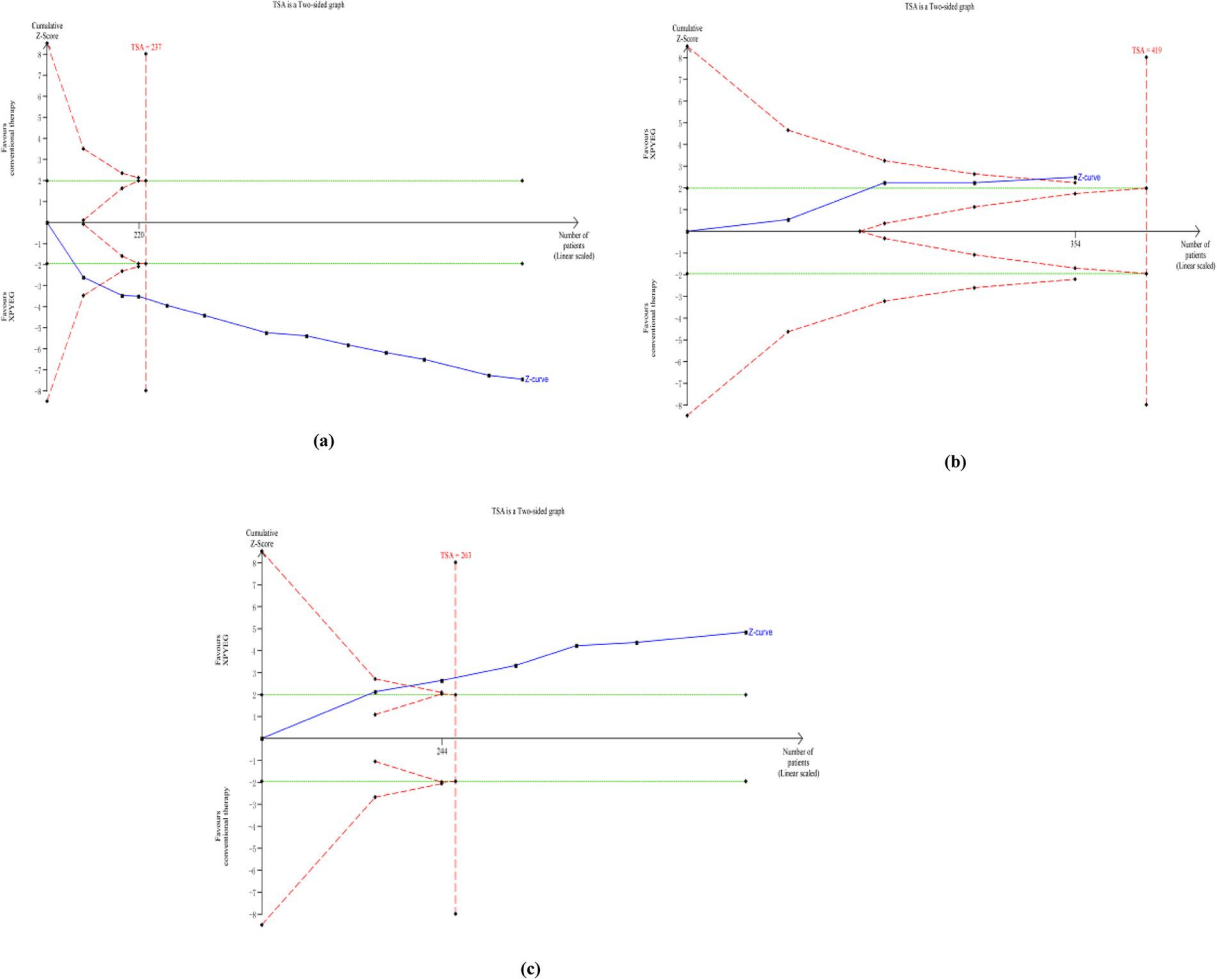
Outcomes of TSA. **(a)** ORR; **(b)** ADRs; **(c)** recurrence rate.

For the ORR, the cumulative Z-curve crossed both the conventional and trial sequential monitoring boundaries, indicating that the required sample size of 237 patients has been met to reach a stable conclusion (Figure 17a). Similarly, the *Z*-curve for the recurrence rate also exceeded both thresholds (Figure 17b). A sample size of 419 patients would be needed to achieve a stable conclusion for adverse events (Figure 17c). These findings demonstrate that the current evidence for both ORR and recurrence rate is robust and reliable, and it is unlikely that future trials will alter these established conclusions.

Cumulative *Z*-curve: The solid blue line represents the trajectory of the test statistic (*Z*-value) for the pooled effect size as the number of studies (samples) progressively increases.

Conventional Boundary: Green solid line, typically a horizontal line fixed at *Z* = ±1.96 (corresponding to a two-tailed *P* = 0.05). This represents the significance threshold for a single test.

Trial Sequential Monitoring Boundary, TSM Boundary: An outward-curving arc established to control the elevated Type I error (false positive) risk arising from multiple interim analyses during the accumulation process.

Futility Boundary: An inward-curving arc. Should the cumulative *Z*-score curve touch this boundary, it indicates that continuing to increase the sample size is unlikely to yield statistically significant results, suggesting a high probability of “futility”. This boundary controls Type II errors (false negatives).

## 4 Discussion

### 4.1 Key findings

This study included 12 RCTs (involving 1,138 patients in total), comprehensively evaluating the efficacy and safety of XPYEG combined with conventional medication for treating NE in children. Results indicate that compared with the control group, compared with the control group, the XPYEG combination therapy achieved a higher clinical response rate. It effectively reduced the frequency of NE-a primary clinical endpoint-while simultaneously increasing estimated bladder capacity and improving several potential mechanism-based indicators, including the arousal threshold during sleep and urine osmolality. Furthermore, it reduced the recurrence rate and the incidence of ADRs, offering a valuable adjunctive treatment strategy to address the multiple challenges posed by conventional medications.

Regarding safety, a lower incidence of overall adverse reactions was observed in the combination group (RR = 0.35). The reported adverse events for XPYEG were predominantly mild gastrointestinal discomfort (e.g., nausea, abdominal pain), which aligns with its known safety profile from the product monograph and previous pharmacological literature. Importantly, the addition of XPYEG did not appear to introduce new or severe safety signals. This favorable tolerability profile, especially within a regimen aimed at long-term management, could positively influence treatment adherence and parental acceptance in pediatric care.

Given the observed clinical benefits, particularly the reduction in enuresis frequency and improvement in bladder capacity, caution should be exercised when interpreting the underlying mechanisms. Given that XPYEG is a multi-component formulation, it is speculated that the observed effects may involve synergistic regulation of the central nervous system and bladder function. For example, preclinical studies on individual Chinese herbal components indicate that triterpenes and volatile oils in *Valeriana jatamansi Jones* exert central inhibitory effects, while the abundant quercetin and flavonoid components in *Emilia sonchifolia* possess anti-inflammatory properties; theoretically, these could respectively help lower the arousal threshold and enhance bladder compliance (31–33); the components of *Pittosporum glabratum Lindl* have antidepressant and sedative effects, which may alleviate emotional disturbances associated with enuresis (34, 35); coumarin compounds found in *Gerbera piloselloides* have also been reported to have antispasmodic effects, which theoretically may help reduce subclinical inflammation of the bladder wall (36). The positive trends in AVP and the cAMP-to-cGMP ratio may reflect secondary neuroendocrine modulatory effects of XPYEG components. However, it must be emphasized that these mechanistic hypotheses are derived from indirect pharmacological literature rather than primary data from this meta-analysis. While existing evidence supports an association between XPYEG and improved clinical efficacy, direct causal pathways remain to be elucidated through well-designed laboratory studies.

### 4.2 Innovation and value

Compared with other systematic reviews published in recent years on the traditional Chinese medicine and modern medical treatments of NE (37, 38), this study holds unique value in the following aspects:

1. Specificity of intervention measures: Focusing on a single, standardized, approved proprietary Chinese medicine (XPYEG) rather than a generalized “traditional Chinese medicine therapy” enhances the clinical applicability and reproducibility of conclusions.
2. Comprehensive outcome measures: Beyond evaluating clinical endpoints such as overall efficacy rate and recurrence rate, it comprehensively analyzed multiple intermediate indicators closely related to the pathophysiology of nocturnal enuresis (e.g., bladder capacity, arousal threshold, AVP, cAMP/cGMP ratios). This provides indirect insights into the mechanism of action across the “symptom-organ-molecular” levels, an aspect largely overlooked in most comparable reviews.
3. Methodological rigor: This study strictly adhered to the PRISMA 2020 guidelines, employing TSA and the GRADE evidence grading system to quantitatively assess the robustness and certainty of evidence. This provides stronger methodological support for the reliability of the conclusions.

### 4.3 Limitations

The limitations of this study are primarily reflected in three aspects: (1) Methodological limitations: Most trials did not report allocation concealment and blinding in detail, potentially introducing a risk of bias; (2) Statistical heterogeneity-enuresis frequency outcome: This study observed high statistical heterogeneity regarding the key outcome of enuresis frequency. In addition to the reasons discussed in the Results section, we believe this may be related to the limited number of studies and inherent clinical differences among the included populations. Although we selected an appropriate meta-analysis model based on the results of heterogeneity tests, the inherent differences in clinical practice may still have some impact on the accuracy of the pooled effect size. (3) Clinical Heterogeneity-Inconsistent Control Group Interventions: The control group interventions included in this study varied. Although statistical heterogeneity was low, clinical heterogeneity still limits the interpretation of the pooled results. The pooled effect size reflects the average adjunctive effect of XPYEG across different control settings; the actual degree of benefit may vary depending on the baseline treatment regimen. Given the small number of studies in each control-type subgroup and the significant differences in intervention mechanisms within these groups, conducting subgroup analyses by control type could lead to misleading conclusions; therefore, such analyses were not performed. The current evidence should be cautiously interpreted to suggest that XPYEG has the potential to provide an adjunctive synergistic effect across various routine pediatric treatment regimens; however, its precise additive effect requires further validation through rigorous studies using desmopressin as the standard control; (4) Geographic concentration of evidence: It is noteworthy that despite a comprehensive systematic search across major international databases (e.g., PubMed, Embase, Cochrane Library), all 12 included RCTs were conducted in China and published in Chinese databases. This reflects the current state of publication for high-quality trials on this specific intervention but results in a geographically concentrated evidence base; (5) Limited generalizability: Consequently, the findings are primarily derived from and applicable to the Chinese pediatric population. Caution is warranted when extrapolating the results to other ethnic, cultural, or healthcare settings, as factors such as genetic background, dietary habits, and acceptance of herbal granules may influence treatment outcomes.

Future research should prioritize international, multicenter, placebo-controlled, and double-blind trials to validate these findings across diverse populations and to strengthen the global evidence base for this complementary therapy.

## 5 Conclusion

In summary, this systematic review and meta-analysis of 12 RCTs (*n* = 1,138) suggests that Xingpi Yanger Granule as an adjunctive therapy may improve overall response, moderately reduce urinary frequency and increase bladder capacity, and decrease recurrence and adverse events. However, due to geographical concentration of the evidence and low-to-moderate quality ratings according to GRADE, current conclusions should be interpreted with caution. Large, well-designed, multicenter randomized controlled trials are needed to validate these potential benefits.

## Data Availability

The original contributions presented in the study are included in the article/Supplementary Material, further inquiries can be directed to the corresponding author.
