# Supplementary material for: Xingpi Yanger Granule combined with conventional medication for the treatment of nocturnal enuresis: a systematic review and meta-analysis of randomized controlled trials
Source: Front Pediatr. 2026 May 12;14:1744259. doi: 10.3389/fped.2026.1744259 (PMC13201404; doi:10.3389/fped.2026.1744259)
Supplement: Supplementary file 1 [file Supplementaryfile1.docx]

**Supplementary material**


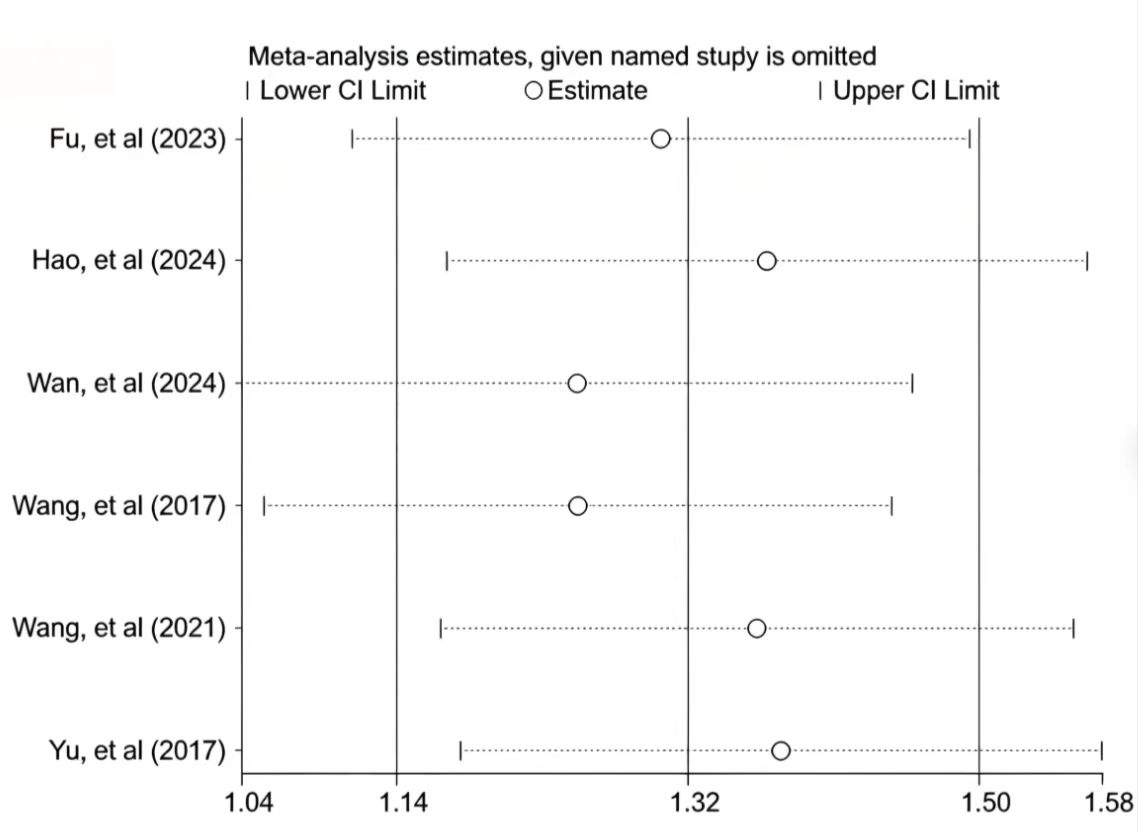


**Figure S1 (a)**

**
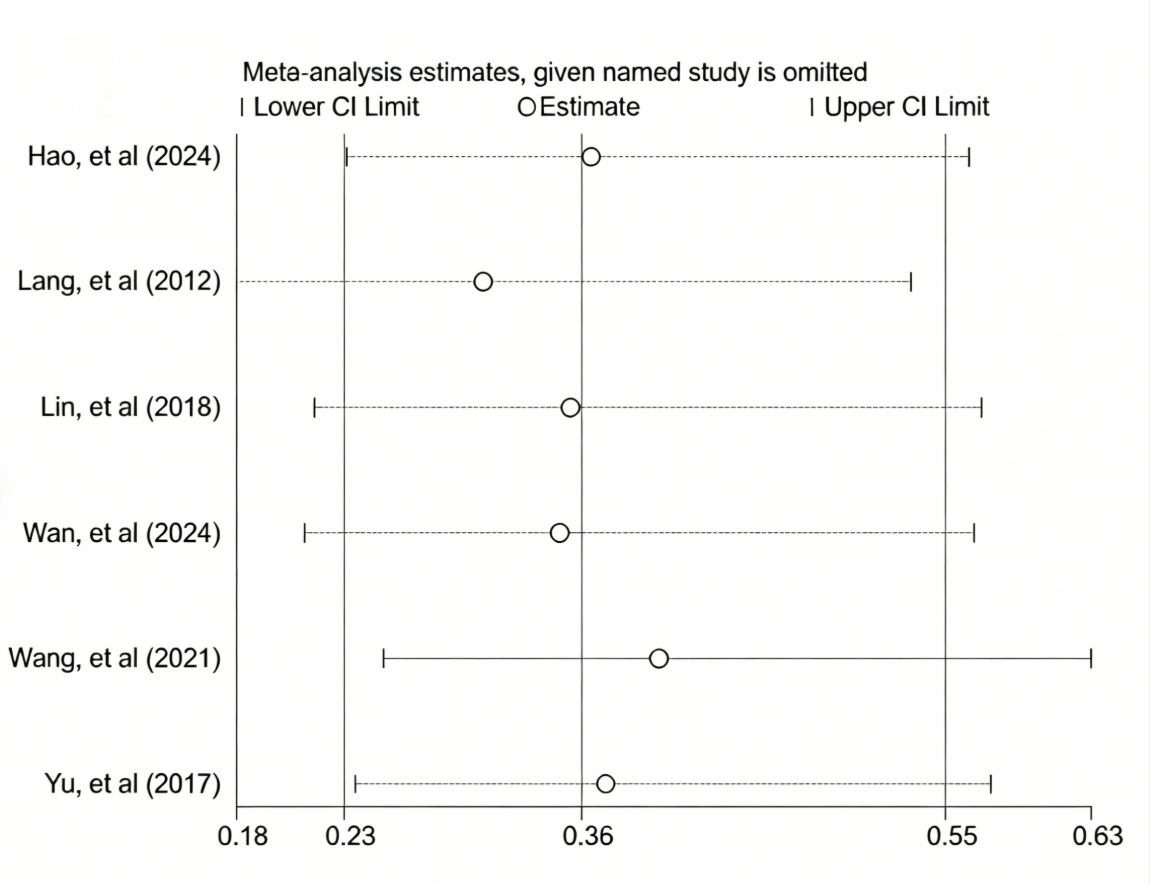
**

**Figure S1 (b)**

**Figure S13:** Sensitivity analyses of primary outcomes: (b) Bladder Capacity; (c) Recurrence Rate.

**Table S1:** Summary of evidence grades based on the GRADE System

**Table S1 (a)**

| Outcome and follow-up | Patients (studies), N | Relative effect (95% CI) | **Absolute effects (95% CI)** | | | Certainty |
| --- | --- | --- | --- | --- | --- | --- |
|  |  |  | **Control group** | **Experimental group** | **Difference** |  |
| ORR | 1138 (12 RCTs) | **RR = 1.25** (1.18 to 1.32) | 719 per 1,000 | **899 per 1,000** (849 to 950) | **180 more per 1,000** (from 129 more to 230 more) | ⨁⨁⨁◯ Moderate^a^ |
| Enuresis Frequency | (4 RCTs) | - | 0 | - | **1.55** (0.66 to 2.43) | ⨁◯◯◯ Very low^a,b,c^ |
| Adverse Drug Reactions | 354 (4 RCTs) | **RR = 0.35** (0.15 to 0.80) | 120 per 1,000 | **42 per 1,000** (18 to 96) | **78 fewer per 1,000** (from 102 fewer to 24 fewer) | ⨁⨁◯◯ Low^a,c^ |
| Recurrence Rate | 656 (6 RCTs) | **RR = 0.30** (0.18 to 0.49) | 210 per 1,000 | **63 per 1,000** (38 to 103) | **147 fewer per 1,000** (from 172 fewer to 107 fewer) | ⨁⨁⨁◯ Moderate^a^ |
| **CI:** confidence interval; **MD:** mean difference | | | | | | |

**Explanations:**

a. Downgraded by one level: None of the studies mentioned the implementation of allocation concealment and blinding methods.

b. Downgraded by one level: I² > 50%.

c. Downgraded by one level: Insufficient number of studies reporting outcomes, n ≤ 5.

**Table S1 (b)**

| Arousal Threshold | Patients (studies), N | Relative effect (95% CI) | **Absolute effects (95% CI)** | | | Certainty |
| --- | --- | --- | --- | --- | --- | --- |
|  |  |  | **Control group** | **Experimental group** | **Difference** |  |
| Arousal Threshold | (4 RCTs) | - | 0 | - | **7.42** (4.54 to 10.3) | ⨁⨁◯◯ Low^a,b^ |
| BVWI | (6 RCTs) | - | 0 | - | **41.31** (36.31 to 46.32) | ⨁⨁⨁◯ Moderate^a^ |
| Urine Osmolarity | (3 RCTs) | - | 0 | - | **16.01** (11.49 to 20.53) | ⨁⨁◯◯ Low^a,b^ |
| Bladder Capacity | (3 RCTs) | - | 0 | - | **44.61** (38.73 to 50.48) | ⨁◯◯◯ Very low^a,b,c^ |
| **CI:** confidence interval; **MD:** mean difference; **RR:** risk ratio | | | | | | |

**Explanations:**

a. Downgraded by one level: None of the studies mentioned the implementation of allocation concealment and blinding methods.

b. Downgraded by one level: Insufficient number of studies reporting outcomes, n ≤ 5.

c. Downgraded by one level: The 95% CI for the combined effect size crosses the dividing line.

**Table S1 (c)**

| Outcome and follow-up | Patients (studies), N | Relative effect (95% CI) | **Absolute effects (95% CI)** | | | Certainty |
| --- | --- | --- | --- | --- | --- | --- |
|  |  |  | **Control group** | **Experimental group** | **Difference** |  |
| cAMP | (3 RCTs) | - | 0 | - | **22.8** (14.42 to 31.17) | ⨁⨁◯◯ Low^a,b^ |
| cGMP | (2 RCTs) | - | 0 | - | **6.37** (4.96 to 7.78) | ⨁⨁◯◯ Low^a,b^ |
| AVP | (6 RCTs) | - | 0 | - | **3.65** (2.02 to 5.28) | ⨁⨁◯◯ Low^a,c^ |
| cAMP/cGMP | (2 RCTs) | - | 0 | - | **6.34** (5.72 to 6.96) | ⨁⨁◯◯ Low^a,b^ |
| **CI:** confidence interval; **MD:** mean difference | | | | | | |

**Explanations:**

a. Downgraded by one level: None of the studies mentioned the implementation of allocation concealment and blinding methods.

b. Downgraded by one level: Insufficient number of studies reporting outcomes, n ≤ 5.

c. Downgraded by one level: The 95% CI for the combined effect size crosses the dividing line.
